# Paclitaxel-loaded polymeric nanoparticles based on α-tocopheryl succinate for the treatment of head and neck squamous cell carcinoma: *in vivo* murine model

**DOI:** 10.1080/10717544.2021.1923863

**Published:** 2021-06-28

**Authors:** Juan Riestra-Ayora, Carolina Sánchez-Rodríguez, Raquel Palao-Suay, Joaquín Yanes-Díaz, Ana Martín-Hita, María Rosa Aguilar, Ricardo Sanz-Fernández

**Affiliations:** a Department otolaryngology, Hospital Universitario de Getafe, Getafe (Madrid), Carretera de Toledo, km 12.500, Getafe, Madrid, Spain; b Faculty of Biomedical and Health Sciences, Universidad Europea de Madrid, Villaviciosa de Odón, Madrid, Spain; c Department of Polymeric Nanomaterials and Biomaterials Institute of Polymer Science and Technology CSIC, Networking Biomedical Research Centre in Bioengineering Biomaterials, and Nanomedicine CIBER-BBN, C/Juan de la Cierva, 3, Madrid, Spain; d Department Pathology, Hospital, Universitario de Getafe, Getafe (Madrid), Carretera de Toledo, km 12.500, Getafe, Madrid, Spain

**Keywords:** Paclitaxel, polymeric nanoparticle, tumor xenograft mouse model, hypopharynx carcinoma squamous cells, tumor growth, apoptosis, angiogenesis

## Abstract

The prognosis of patients with recurrent or metastatic head and neck squamous cell cancer (HNSCC) is generally poor. New treatments are required to supplement the current standard of care. Paclitaxel (PTX), an effective chemotherapeutic for HNSCC, has serious side effects. A polymeric nanocarrier system was developed for the delivery of PTX to improve HNSCC treatment. This study aimed to evaluate the antitumor efficacy of PTX-loaded polymeric nanoparticles based on α-TOS (PTX-NPs) administered by direct intratumoral injection into a Hypopharynx carcinoma squamous cells (FaDu) tumor xenograft mouse model. The nanocarrier system based on block copolymers of polyethylene glycol (PEG) and a methacrylic derivative of α-TOS was synthesized and PTX was loaded into the delivery system. Tumor volume was measured to evaluate the antitumor effect of the PTX-NPs. The relative mechanisms of apoptosis, cell proliferation, growth, angiogenesis, and oxidative and nitrosative stress were detected by Western blotting, fluorescent probes, and immunohistochemical analysis. The antitumor activity results showed that compared to free PTX, PTX-NPs exhibited much higher antitumor efficacy and apoptosis-inducing in a FaDu mouse xenograft model and demonstrated an improved safety profile. Ki-67, EGFR, and angiogenesis markers (Factor VIII, CD31, and CD34) expression were significantly lower in the PTX-NPs group compared with other groups (*p* < .05). Also, PTX-NPs induced oxidative and nitrosative stress in tumor tissue. Direct administration of PTX-loaded polymeric nanoparticles based on α-Tocopheryl Succinate at the tumor sites, proved to be promising for HNSCC therapy.

## Introduction

Approximately 650,000 new cases of head and neck squamous cell carcinoma (HNSCC) are diagnosed each year worldwide and despite modern treatment approaches, more than half of all diagnosed patients eventually experience local, regional, or metastatic recurrence (Denaro et al., 2014). Especially for patients with locoregional recurrences or for patients with severe comorbidities, such as elderly patients, they cannot undergo the injury caused by surgical treatment. In most cases with inoperable and metastatic disease, two options for palliative treatment remain local radiotherapy or systemic chemotherapy, to get improvements in survival and quality of life (Colevas, 2006; Bakker et al., 2018). The dose of a chemotherapeutic agent used for systemic chemotherapy delivered might be toxic on the organism, which requires a good performance status of the patients. An alternative approach to systemic administration of drugs for the treatment of localized tumors could be through loco-regional administration. This can be achieved by direct intratumoral injection such targeted delivery would be expected to provide a high local concentration of the anticancer drug, reducing systemic drug levels and thereby decreasing the incidence of side effects commonly observed with systemic therapy (Lee et al., 2002; Chvapil, 2005). Palliative intratumoral injection chemotherapy is clinically used in treating lung cancer, extremity melanoma, dysphagia caused by carcinoma, and recurrent HNSCC (Xie et al., 2007; Gimbel et al., 2008; Ramakrishnaiah et al., 2014; Li et al., 2016).

Paclitaxel (PTX), a potent chemotherapeutic antitumor agent, has been used in the treatment of patients with locally advanced HNSCC (Bucci et al., 2004; Abitbol et al., 2005). PTX is a taxane that stabilizes microtubules and, consequently inhibits mitosis. The major limitation of PX is its low water solubility. Thus, the commercial formulation is a concentrated solution of PTX in an organic mixture of dehydrated alcohol and polyoxyethylated castor oil (Cremophor-EL) at a ratio of 1:1 (v/v) (Singla et al., 2002). However, severe toxicities of the marketed formulation of PTX, Taxol®, are attributed to the presence of Cremophor-EL, such as bone marrow suppression, hypersensitivity, neurotoxicity, and nephrotoxicity reactions (Singla et al., 2002). Occasionally, these side effects are too severe, forcing the termination of the treatment even in patients who have no alternative left. Efforts to eliminate these problems have focused on developing new drug delivery systems for PTX, such as microspheres, microparticles, and nanoparticles, that achieve site-specific delivery, prolong action periods and improve patient compliance (Nsereko and Amiji, 2002; Wu et al., 2015; Yang et al., 2017). Encapsulation of active components into carriers become a hopeful method of preventing drug degradation, increasing drug bioavailability, decreasing drug toxic effects, achieving specific targeting, and controlling drug release (Pope-Harman et al., 2007).

In this sense, our group has synthesized nanovehicles of a methacrylic derivative of α-tocopheryl Succinate (α-TOS), MTOS, and copolymerized forming amphiphilic macromolecules that were able to self-assemble in aqueous media forming surfactant-free nanoparticles (NPs) (Palao-Suay et al., 2015; Palao-Suay et al., 2016). The hydrophilic segment of the copolymers was based on polyethylene glycol (PEG) and the hydrophobic segment incorporated MTOS: PEG-/B/-POLYMTOS (71:29) (Palao-Suay et al., 2016). The hydrophilic nature of PEG molecules on the surface of the nanoparticles avoids opsonization and recognition by the reticuloendothelial system, provides high circulation times, and pronounced enhanced permeation and retention effect (Jokerst et al., 2011). Experimental evidence indicated that vitamin E succinate (tocopheryl succinate, α-TOS) is one of the most effective anticancer and antiangiogenic compounds of the vitamin E family. α-TOS has been described as a mitochondrially targeted anticancer compound (mitocan) (Dong et al., 2009; Duhem et al., 2014). The α-TOS actions on cancer cells produce high levels of reactive oxygen species (ROS) that activate the intrinsic apoptosis cascade exposure and therefore induce cell death (Dong et al., 2009). α-TOS has been conjugated in different drug delivery systems and due to the hydrophobic nature, improves the drug loading efficiency of the system (Liang et al., 2012; Li et al., 2014). We have recently shown that PEG-/B/-POLYMTOS loaded with α-TOS decreased cell viability in an *in vitro* model of breast cancer (MDA-MB-453 cells) (Palao-Suay et al., 2016). Besides, in previous studies of our group, in hypopharynx carcinoma squamous cells (FaDu cells) we verified the antitumor and antiangiogenic effect mediated by ROS of α-TOS-based polymeric nanoparticles (Palao-Suay et al., 2016; Sánchez-Rodríguez et al., 2018).

For that reason, this work aimed to evaluate antitumor effects of amphiphilic nanoparticles as drug delivery systems of PTX using PEG as a hydrophilic domain and MTOS as a hydrophobic block, injected intratumorally in a murine xenotransplantation model of HNSCC.

## Material and methods

### Synthesis of the copolymers and nanoparticles

The methacrylic derivative of α-TOS (MTOS), the PEG macro-CTA agent (4-cyano-4-[(dodecyl sulfanyl thiocarbonyl)sulfanyl)pentanoic acid was conjugated to PEG with Mn of 8 kDa) and PEG-/B/-POLYMTOS with a copolymer molar composition of PEG: MTOS 71:29 (from now on PEG-71) were obtained as previously described (Palao-Suay et al., 2016). Briefly, the block copolymer was synthesized by RAFT polymerization of MTOS and PEG macro-CTA agent in anhydrous 1,4-dioxane at 70 oC for 5 h. PEG-71 was purified by dialysis against distilled water for 72 h, and isolated by freeze-drying.

Self-assembled NPs were prepared by self-organized precipitation (SORP). Specifically, PEG-71 was dissolved in 1,4-dioxane at 10 mg/mL. Moreover, an aqueous solution of NaCl (100 mM) was incorporated drop by drop over the organic phase under constant magnetic stirring to obtain a final polymer concentration of 5 mg/mL PTX (10% w/w respect to the polymer) was also added to the organic phase to prepare NPs that entrapped this hydrophobic molecule in their inner core. After the precipitation, milky NP dispersions were dialyzed against NaCl for 72 h to remove organic solvents and unloaded the drug. Afterwards, each NP suspension was sterilized by filtration through 0.22 µM polyethersulfone membranes (PES, Millipore Express®, Millex GP, Burlington, MA, USA) and stored at 4 °C. To calculate the encapsulation efficiency, PTX-NPs were freeze-dried to eliminate the aqueous phase. Afterwards, PTX entrapped in the NPs was quantified by absorbance spectroscopy. Specifically, PTX loaded NPs (5 mg/mL) were dissolved in ethanol and their absorbance was measured at 227 nm using a Perkin Elmer Lambda 35 UV/VIS spectrophotometer.

The particle size distribution of the unloaded and PTX-NP suspensions was determined by dynamic light scattering (DLS) using a Malvern Nanosizer NanoZS Instrument equipped with a 4mW He-Ne laser (λ = 633 nm) at a scattering angle of 173°. Measurements of NP dispersions were performed in square polystyrene cuvettes (SARSTEDT, Nümbrecht, Germany), and the temperature was kept constant at 25 °C. The zeta potential was determined for NPs formulations at 0.1 mg/mL concentration containing 10 mM NaCl and using laser Doppler electrophoresis (LDE).

Paclitaxel suspension control was provided by the University Hospital of Getafe (Madrid, Spain). Paclitaxel injection was diluted prior in 0.9% Sodium Chloride to a final concentration of 1 mg/mL. The solutions were administrated immediately after being visually inspected for particulate matter and discoloration.

### Cell culture

Hypopharynx carcinoma squamous cells (FaDu) from ATCC (Manassas, VI, USA) were cultured in Dulbecco’s modified Eagle’s medium (DMEM) containing 10% fetal bovine serum (FBS; Gibco, Thermo Scientific, MA, USA).) and 1% penicillin/streptomycin (Sigma-Aldrich, San Luis, MO, USA). Cell cultures were incubated at 37 °C and 5% CO2.

### Establishment of HNSCC xenograft subcutaneous tumor model

Female Athymic Foxn1 nude mice (supplied by ENVIGO Laboratories, IN, USA) weighing 18–20 g were acclimatized for 7 days before the experiment and allowed free access to a standard diet and water with the maintained condition of 22–25 °C temperature and 50% relative humidity. All care and handling of animals were performed with the approval of the Committee of the Ministry of Environment of the Autonomous Community of Madrid, reference number: PROEX 267/14.

For this experiment, the female Foxn1 nude mice were injected subcutaneously with 0.1 mL FaDu cell suspension (5 × 10^6^ cells) with 1:1 vol. basement membrane matrix, Matrigel® (Corning, NY, USA) into the dorsal skin. When tumor size reached ∼150–250 mm^3^, tumor-bearing mice were allocated randomly to drug administration groups.

The animals were divided into four groups (*n* = 7) as follows: control group (C); unloaded MTOS nanoparticles group (Ø-NP); PTX group (PTX); and PTX-loaded MTOS nanoparticles group (PTX-NP).

The mice in the Control group received an injection of 0.9% saline solution. The Ø-NP group is administered unloaded MTOS nanoparticles (1 mg/ml). The animals in the PTX-NP and PTX groups were administered at a dose of 5 mg/kg respectively; PTX-NP was dissolved and diluted in 5% glucose and PTX in 0.9% saline (1 mg/ml). Mice were administered intratumorally once a week for 3 weeks (5 mg/kg, qw x 3).

Drugs were injected intratumorally by puncturing the entire tumor so that drug was distributed homogeneously. The tumor volume was calculated using the formula: tumor volume (mm^3^) = a x b (where a is the longest tumor diameter and b is the shortest tumor diameter). At the end of the experiment (day 27), all mice were sacrificed and the tumors were extracted. Subsequently, the tumor was divided into two pieces, a part was included in paraffin blocks for histological and immunohistological evaluation; the other part of the tumor proteins was extracted for later analysis.

### Western blot

Tumors were harvested from the tumor-bearing mice after 3 weeks of treatment and then lysed in a buffer T-Per (Pierce, Thermo Scientific, MA, USA).) with Complete™ Protease Inhibitor Cocktail (Roche, Basel, Switzerland). Total protein extracts (40 µg) were resolved by sodium dodecyl sulfate polyacrylamide gel electrophoresis and transferred to a nitrocellulose membrane (Bio-Rad, CA, USA). After blocking the membranes, immunodetection was performed using an antiserum specific for EGFR (1/1000), Annexin-V (1/500), Nitrotyrosine (1/250), and β-actin (1/2000, used as a loading control) of Abcam (Cambridge, UK) followed by incubation with rabbit peroxidase-conjugated secondary antibody (1:10,000 dilution, Abcam). Immunoreactive bands were visualized using enhanced chemiluminescence detection by the system SuperSignal West Pico Chemiluminescent Substrate (Thermo Scientific, MA, USA). Densitometric analysis of the film was performed using a MultiImageTM Light Cabinet Filter Positions imaging densitometer in transmittance mode and analyzed using AlphaFaseFC (Fluor Chem) software (Alpha Innotech Corporation, CA, USA).

### Histology

Tumor tissue was fixed in paraformaldehyde (4%) in phosphate-buffered saline (PBS) (2 M, pH 7.36). Briefly, tumor tissue samples were then dehydrated through a series of ethanol washes (50%, 90%, and 100% in dH_2_O) and incubation in Histo-Clear (Thermo Scientific, MA, USA). Following incubation in paraffin wax for 1 h at 60 °C, samples were embedded in wax blocks. The paraffin-embedded tumor was cute in consecutive 5 μm thick sections and was rehydrated through Histo-Clear, graded ethanol, and dH_2_O before staining with Hematoxylin and Eosin. The samples were visualized by microscopy Olympus BX51 (Tokyo, Japan).

### Indirect immunofluorescence

Paraffin-embedded tumor tissue sections (5 µm thick) were mounted on polysine-coated glass slides. In brief, sections of tumor tissues were irradiated with microwave in Trilogy™ (Cell Marque, Sigma-Aldrich, San Luis, MO, USA) solution that deparaffinized, rehydrate and unmask the tissue samples for 10 min to maximum power. After, blocked for 2 h at 37 °C and incubated overnight at 4 °C with antibodies: (i) anti-factor VIII (dilution 1:100; Abcam); (ii) anti-EGFR (dilution 1:50; Abcam); anti-annexin V (dilution 1:50; Abcam), as the primary antibodies (Cambridge, UK). Following washes, sections were incubated with the secondary Alexa Fluor 488-conjugated goat anti-rabbit antibody or Alexa Fluor 546-conjugated goat anti-rabbit antibody (dilution 1:250; Molecular Probes, Invitrogen, Thermo Scientific, MA, USA) for 45 min at 37 °C. Next, sections were incubated with 4′,6-diamidino-2-phenylindole (DAPI, 300 nM) for 5 min at 37 °C, reactive with fluorescent blue, marking the interlayer between DNA base pairs of cell nuclei. After further washing in PBS, sections were mounted and visualized by microscopy. The specificity of the immunostaining was evaluated by the omission of the primary antibody (negative controls). Tissue sections were scored for fluorescence using an Olympus BX51 (Tokyo, Japan) (To fluorescence microscope and the specificity was evaluated by the omission of the primary antibody. ImageJ, a free image-processing program, was used for quantitative analysis of the image.

### Immunohistochemical (IHC) staining

The paraffin-embedded tissue sections (5 µm) were prepared with Trilogy™ solution (Cell Marque, Sigma-Aldrich, San Luis, MO, USA) and subsequently incubated with antibodies: anti-Ki-67; anti-p53; anti-CD31; anti-CD34, and anti-bcl2 (DAKO, Santa Clara, CA, USA) at 4 °C overnight followed by EnVision + System- HRP Kit (DAKO Santa Clara, CA, USA) following the manufacturer's instructions. Tumor sections were mounted and visualized by microscopy Olympus BX51 (Tokyo, Japan). ImageJ was used for quantitative analysis of the image.

### Superoxide anion detection

Tumor sections (5 µm thick) were mounted on polysine coated glass slides and were incubated for 90 min at 37 °C with the fluorescent probe dihydroethidium (DHE, 4 µmol/l; Calbiochem, Burlington, MA, USA). In the presence of superoxide anions, DHE is oxidized to ethidium, which intercalates with DNA, and yields bright red fluorescence. Thereafter, sections were incubated with DAPI (300 nM) for 5 min at 37 °C. DAPI staining of cell nuclei helps detect true DHE staining (present in the nucleus) versus nonspecific staining. After washing, sections were mounted and visualized by fluorescence microscopy (Olympus BX51, Tokyo, Japan). The specificity of the staining was evaluated by the omission of the dye (negative controls). ImageJ was used for quantitative analysis of the image.

### Statistical analysis

Results were expressed as a mean ± standard deviation (SD). Statistical analysis was performed using one-way analysis of variance (ANOVA) following by Bonferroni post hoc analysis with computer software IBM SPSS 21.0 (IBM Inc., Armonk, NY; USA). A p-value < 0.05 and *p* < 0.01 were considered significant and very significant, respectively.

### Ethics approval

All care and handling of animals were performed with the approval of the Committee of the Ministry of Environment of the Autonomous Community of Madrid, reference number: PROEX 267/14. All institutional and national guidelines for the care and use of laboratory animals were followed.

## Results

### Nanoparticle characterization: PTX encapsulation

Polymeric NPs were successfully prepared and characterized and the results are summarized in Table 1. The hydrodynamic diameters were 135 and 125 nm for unloaded and PTX loaded NPs, respectively, with unimodal size distributions, low PDI values, and zeta potentials slightly negative, indicating an almost neutral charge of the NPs surface. Additionally, PTX was efficiently entrapped in the inner core of the NPs with an EE of 72.1%.

**Table 1.** Most relevant characteristics of unloaded and PTX loaded NPs: encapsulation efficiency (EE), Hydrodynamic diameter (Dh, by intensity), size distribution width (Wd), polydispersity index (PDI); and zeta potential values (ζ), measured by DLS and LDE respectively.

NP sample	Polymer + drug	EE (%)/PTX (mg/ml)	*D*_h_ (nm)	*W*d (nm)	PDI	ζ (mV)
∅-NP	NP-71	--	134.9 ± 6.5	31	0.079 ± 0.012	–2.38
PTX-NP	NP-71 + PTX	72.1/0.36	125.2 ± 9.7	49	0.090 ± 0.011	–1.24

### PTX-NP inhibit tumor growth in HNSCC tumor xenografts

**Figure 1.**
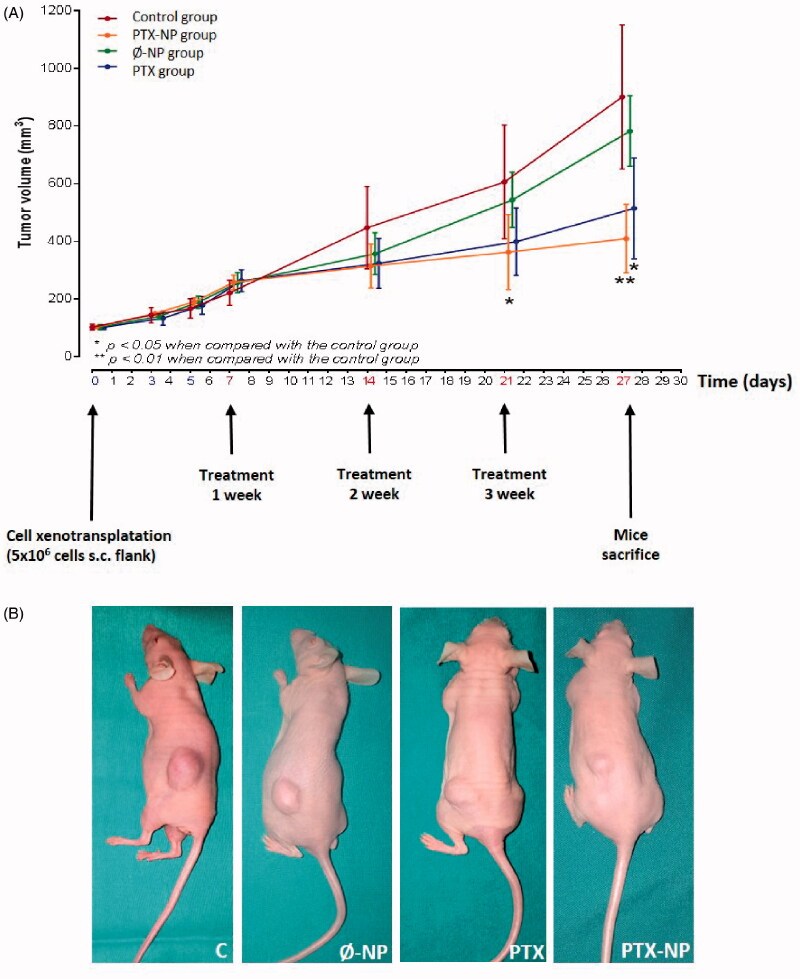
*In vivo* antitumor activity of PTX-NP in tumor-bearing nude mice. (A) FaDu cells were implanted in the nude mice on day 0, and the treatment was started on day 7 when the tumor volume reached ∼150–250 mm^3^. The treatment involved: C (control); Ø-NP (unloaded NPs, 1 mg/ml; PTX (Paclitaxel, 5 mg/kg); and PTX-NP (Paclitaxel loaded NPs, 5 mg/kg) on days 7, 14 and 21. Data are presented as the mean ± SD per group measured on the indicated days after treatment (*n* = 9). Each data point is the mean value (SD) of volume tumors. *p < .05, **p < .01 compared to the control group. (B) Photographs of representative mice and tumors treated with drugs are shown.

The antitumor effect of PTX-NP was evaluated with transplanted FaDu cell xenografts in nude mice. At the end of the experiment (treatment dose once a week for 3 weeks), the tumor volumes in various groups were measured and are reported in Figure 1(A). Compared to the Control group, the PTX (*p* < 0.05) and PTX-NP (*p* < 0.01) groups significantly reduced tumor volume. The results revealed that administration of PTX-NP induced the best tumor growth inhibition rate (410 mm3) compared to the control group (901 mm3), Ø-NP group (783 mm3), and PTX group (515,1 mm3). These results indicate that the efficacy of PTX-loaded nanoparticles is greater than PTX suspension although, the difference was not significant. Figure 1(B) shows representative photographs of tumor size in nude mice. Growth differences were observed between the different treatment groups.

### Anti-proliferative effects of PTX-NPs in HNSCC model

**Figure 2.**
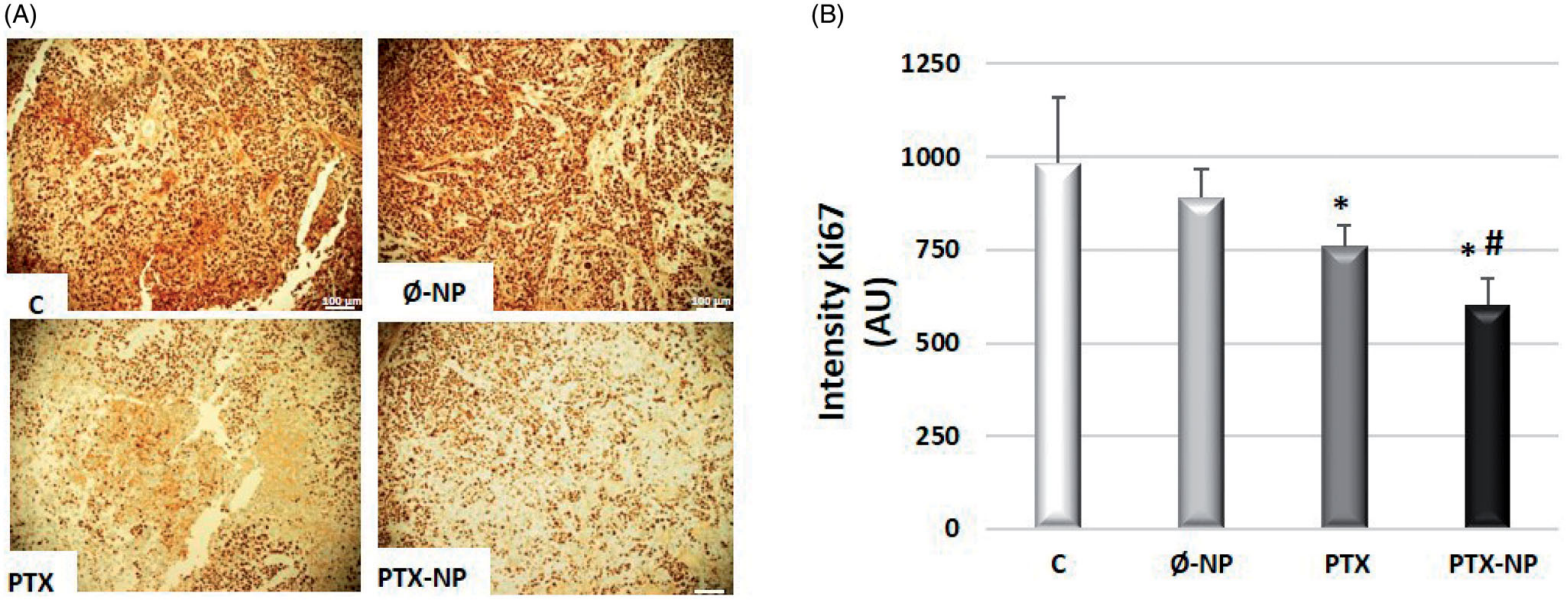
Anti-proliferative effects of PTX-NPs in HNSCC model. (A) Representative micrographs of immunostaining with Ki67 of all treatment groups. Cell nuclei are identified with immunoreactivity for Ki67 marked in brown. 100 mm scale bar. Capture of representative images was performed randomly, blindly; Mice treated with: C (control); Ø-NP (unloaded NPs, 1 mg/ml; PTX (Paclitaxel, 5 mg/kg); and PTX-NP (Paclitaxel loaded NPs, 5 mg/kg) for 21 days; (B) Quantification of immunostaining for Ki67 measured by the ImageJ program. The diagrams for Ki67 represent the mean ± SD of arbitrary units (AU) *p < .05 versus C and Ø-NP groups; ^#^*p* < .05 versus group PTX. (*n* = 20).

Immunohistochemical analysis of Ki-67 staining revealed a greater inhibition of proliferating cells in the PTX-NP treated mice than the control and unloaded-nanoparticle groups [Figure 2(A)]. Besides, there was a statistically significant difference in mean Ki-67 expression between PTX-NP and PTX groups (*p* < 0.05). The expression rate of Ki67 in the PTX-NP group was 575,8 ± 9 (AU), which was significantly lower compared with the other treatments [PTX, 759,2 ± 17; Ø-NP, 898,6 ± 13; and C, 982,34 ± 14; all *p* < 0.05; Figure 2(B)].

### PTX-NP downregulate EGFR expression in the HNSCC model

**Figure 3.**
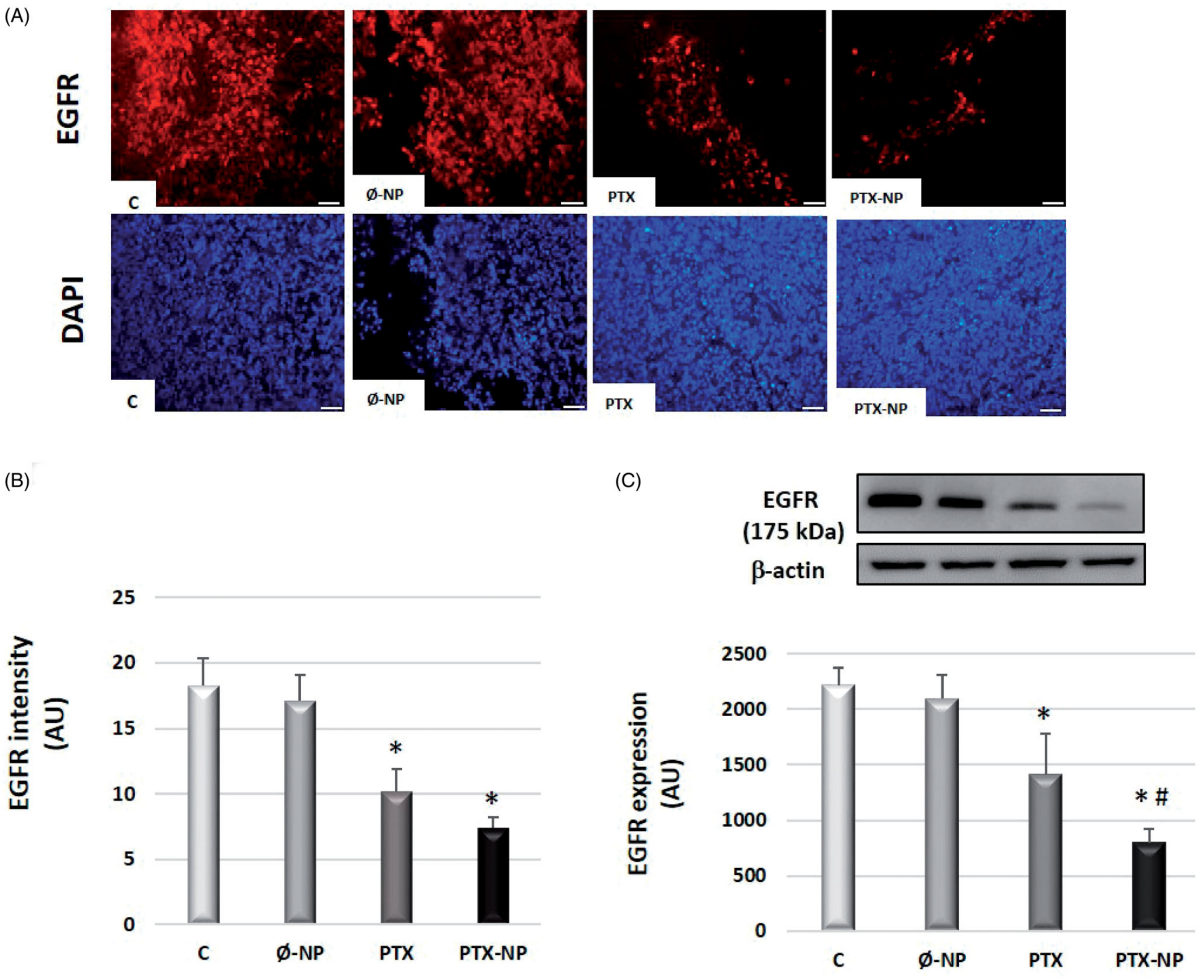
PTX-NP downregulates EGFR expression in the HNSCC model. (A) Fluorescence micrographs of representative immunostaining of EGFR-Alexa Fluor546 conjugate in tumor tissue (red) and 4´,6-diamidino-2-phenylindole dihydrochloride (DAPI) (blue) (x40), after treatment C (control); Ø-NP (unloaded NPs, 1 mg/ml; PTX (Paclitaxel, 5 mg/kg); and PTX-NP (Paclitaxel loaded NPs, 5 mg/kg) for 21 days. Scale bar 100 mm. (B) EGFR immunofluorescence intensity was analyzed by ImageJ software. (C) EGFR protein expression was determined by Western blotting analysis. β-actin was used as a loading control. The diagrams for EGFR (B and C) represent the mean ± SD of arbitrary units (AU). *p < .05 versus C and Ø-NP groups; ^#^*p* < .05 versus group T. (*n* = 20).

EGFR expression was measured by immunofluorescence and western-blot analysis (Figure 3A-B and 3 C, respectively). The PTX-NP group had the lowest EGFR expression (7.4 ± 0.9 AU), while in the C group was 18.27 ± 2 AU (*p* < .05), in the Ø-NP group was 17.1 ± 1.9 AU (*p* < .05) and in the PTX group was 10.2 ± 1,7 AU (*p* < .05). All p values were obtained compared to the PTX-NP group [Figure 3(B)]. Similar results were obtained by western-blot analysis [Figure 3(C)].

### PTX-NP induces apoptosis in the HNSCC tumor model

**Figure 4.**
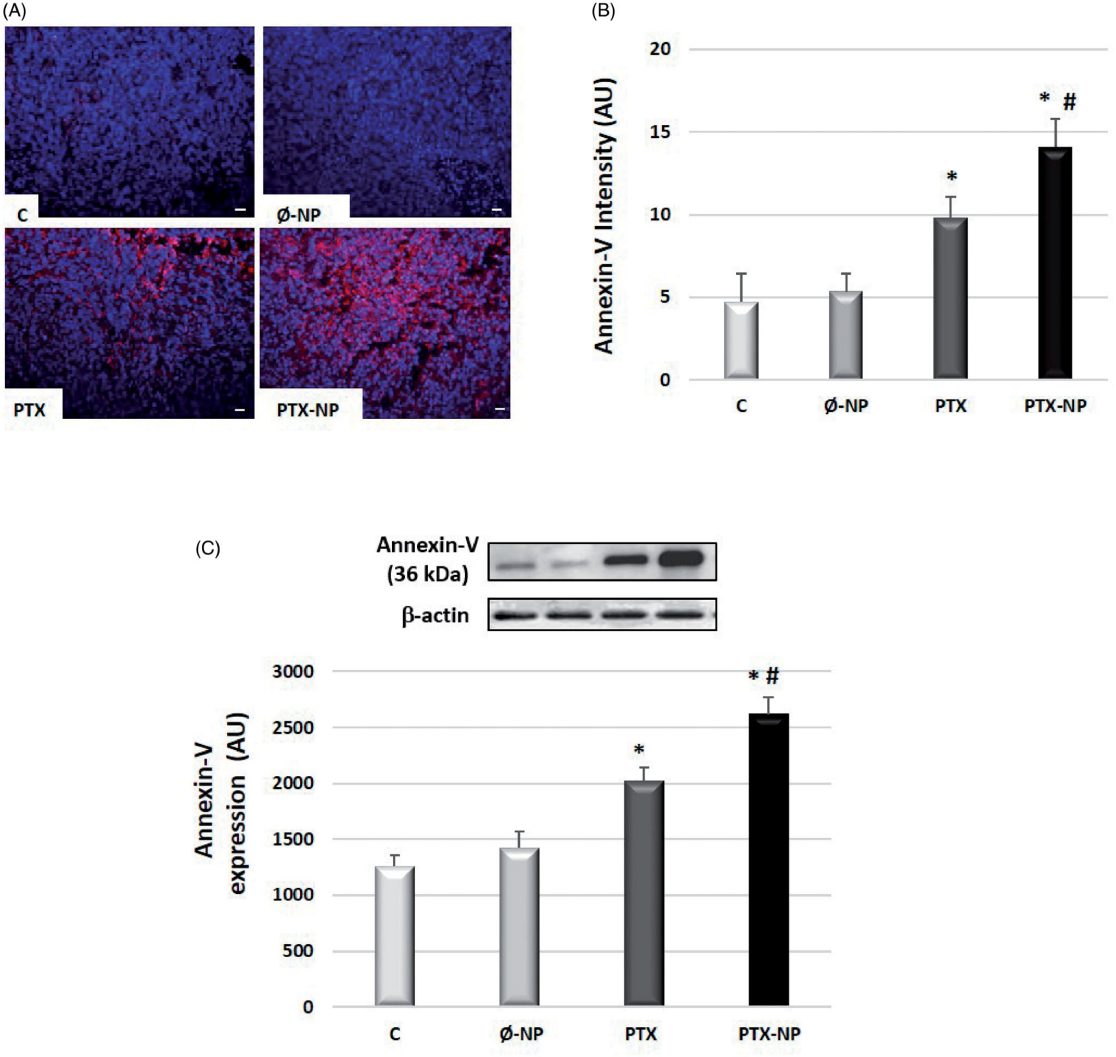
PTX-loaded nanoparticles induce apoptosis in HNSCC *in vivo* model. Treatment groups: C (control); Ø-NP (unloaded NPs, 1 mg/ml; PTX (Paclitaxel, 5 mg/kg); and PTX-NP (Paclitaxel loaded NPs, 5 mg/kg) for 21 days. (A) Representative immunostaining of annexin V in tumor tissue. The cytoplasm shows annexin-positive staining (red) characteristic of apoptotic cells (x40). Scale bar 100 mm. (B) Representative western blot of annexin-V protein expression levels and densitometric analysis. β-actin was used as a loading control. Data represent the mean ± SD of arbitrary units (AU). *p < .05 versus C and Ø-NP groups; ^#^*p* < .05 versus group PTX. (*n* = 20).

Annexin-V analysis was performed to determine cellular apoptosis. As shown in Figure 4(A,B), immunofluorescence analysis, PTX-NP group apoptotic rate increased from 9,81 to 14,07 (AU) compared with the PTX group (*p* < .05), while the control group was 4,73 (AU) (*p* < .05).

Additionally, we performed western-blot experiments to verify PTX-NP induction of apoptosis in HNSCC *in vivo* model. As it is shown in Figure 4(C), annexin-V expression significantly increased using PTX-NP and PTX concerning control (*p* < .05). Particularly, the induction of apoptosis was remarkable in the case of PTX-NP group compared to C, Ø-NP, and PTX groups (*p* < .05).

### Effects of PTX-loaded nanoparticles on the expression of apoptotic markers

**Figure 5.**
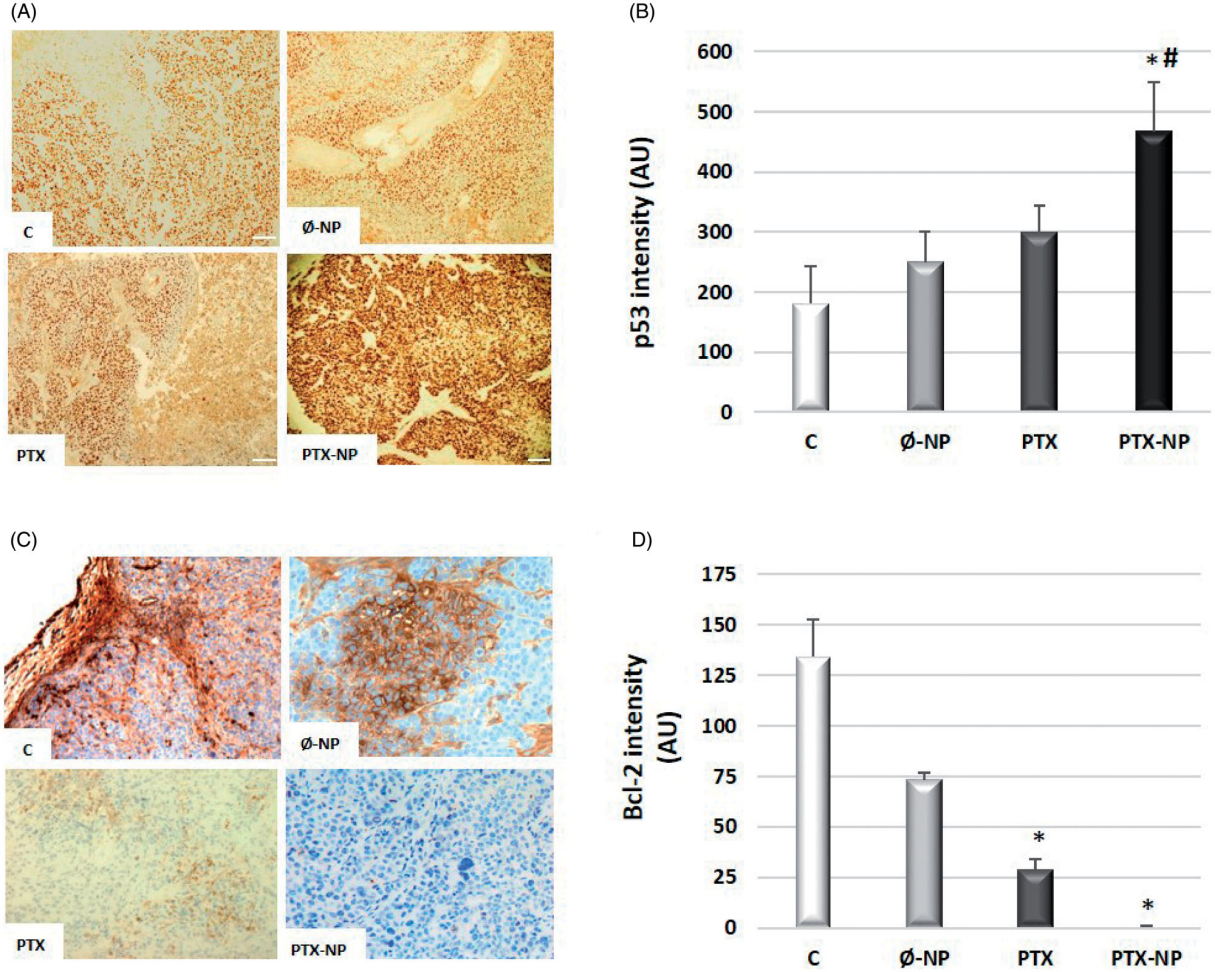
Effects of PTX-loaded nanoparticles on the expression of apoptotic markers. Representative images of tissue slices stained for p53 (A) and Bcl-2 (C) expressions in treated animal with: C (control); Ø-NP (unloaded NPs, 1 mg/ml; PTX (Paclitaxel, 5 mg/kg); and PTX-NP (Paclitaxel loaded NPs, 5 mg/kg) for 21 days. Quantification of p53 (B) and Bcl-2 (D) positive cells according to image analysis by the ImageJ software. Data represent the mean ± SD of arbitrary units (AU). *p < .05 versus C and Ø-NP groups; ^#^*p* < .05 versus group PTX. (*n* = 20).

P53 and Bcl-2 proteins were analyzed by immunohistological analysis (Figure 5). As shown in Figure 5(A,B), p53 protein levels were increased in the PTX-NP group relative to the C, Ø-NP, and PTX groups, which was significant versus the C group (*p* < .05). The expression levels of Bcl-2 were dramatically decreased in the tumors treated with PTX-NP as compared with others groups (*p* < .05) [Figure 5(C,D)]. These findings fit well with the decrease in proliferation of tumor cells and increases in cell apoptosis in mice treated with PTX-NP in the HNSCC model.

### PTX-NP increase oxidative and nitrosative stress levels in tumor tissue

**Figure 6.**
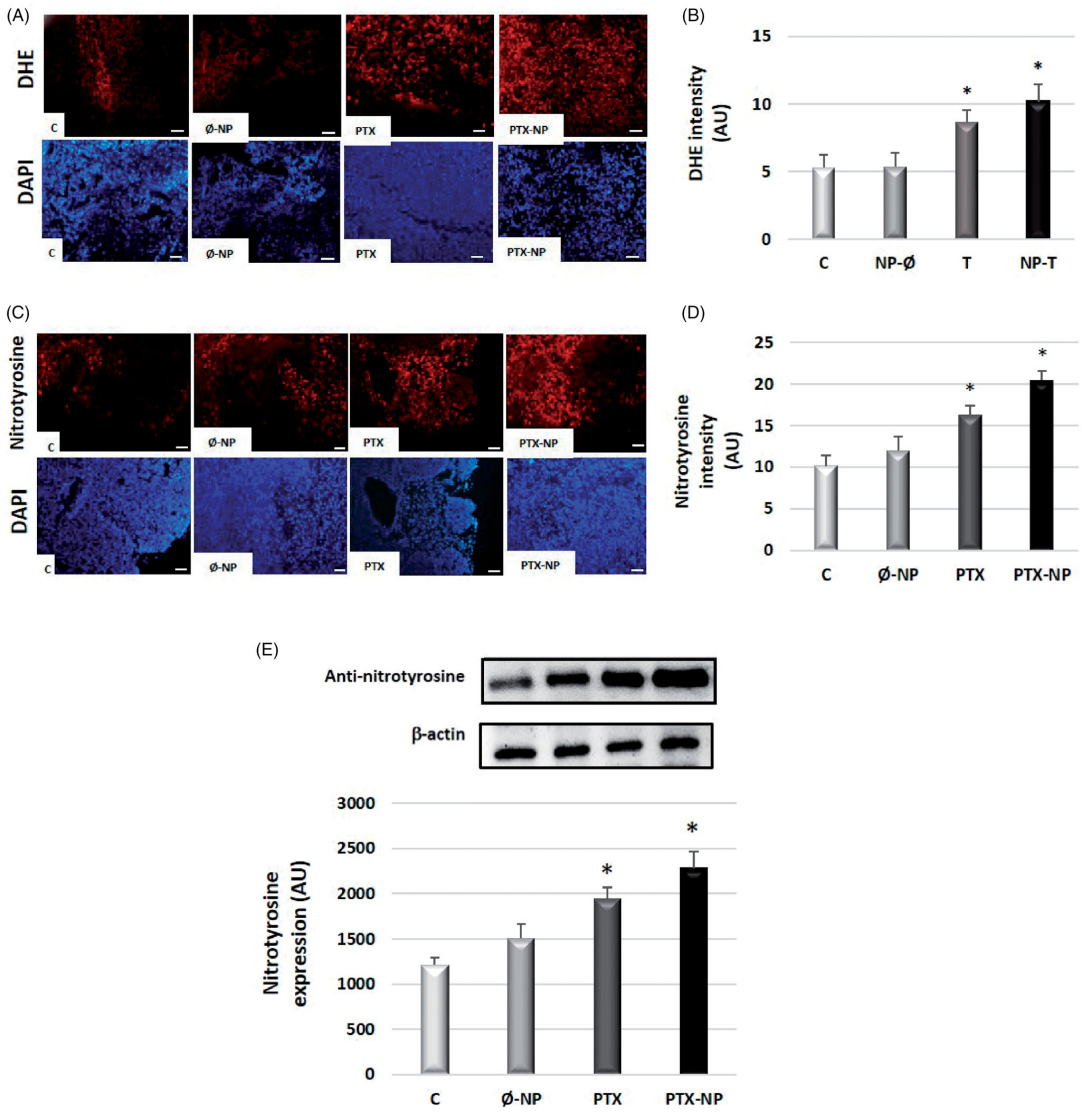
Paclitaxel-loaded nanoparticles increase levels of oxidative and nitrosative stress in the HNSCC mice model. Experimental groups: C (control); Ø-NP (unloaded NPs, 1 mg/ml; PTX (Paclitaxel, 5 mg/kg); and PTX-NP (Paclitaxel loaded NPs, 5 mg/kg for 21 days. (A) Superoxide anion detection with the fluorescent probe dihydroethidium in red (DHE) and DAPI in blue dye in tumor tissue, (x40). Scale bar 100 mm. (B) Quantification of fluorescence intensity for DHE by ImageJ software. (C) Representative immunostaining of nitrotyrosine in tumor tissues. The cytoplasm shows nitrotyrosine-positive staining (red), (x40). Scale bar 100 mm. (D) Nitrotyrosine immunofluorescence intensity was analyzed by ImageJ software. (E) Quantification of nitrotyrosine detected in the blots using densitometry analysis. Data represent the mean ± SD of arbitrary units (AU). *p < .05 versus C and Ø-NP groups; (*n* = 20).

Oxidative and nitrosative stress levels were enhanced with PTX-NP treatment in the tumor. Intracellular prooxidant production (presumably superoxide anion, O2-.) in tumor tissue was determined by measuring DHE oxidation. PTX-NP and PTX groups significantly increased DHE oxidation in tumor, 10.1 ± 1.3 and 8.7 ± 0.86 (AU) respectively, compared with control (5.3 ± 0.9 AU) (*p* < .05), and Ø-NP (5.35 ± 1 AU) groups [Figure 6(A,B)].

An anti-nitrotyrosine-specific antibody was used to study the presence of nitrotyrosine, a footprint of peroxynitrite formation (a type of reactive nitrosative species, RNS). To quantify nitrotyrosine levels in tumor tissue, we performed an immunofluorescence and western blot analysis. As shown in Figure 6(C–E), the expression levels of nitrotyrosine increased significantly in PTX-NP and PTX groups compared to the control group (*p* < .05). These data suggest that PTX-NP induces oxidative and nitrosative stress in HNSCC murine model characterized by superoxide anion and peroxynitrite production.

### PTX-NP inhibits angiogenesis in the HNSCC tumor model

**Figure 7.**
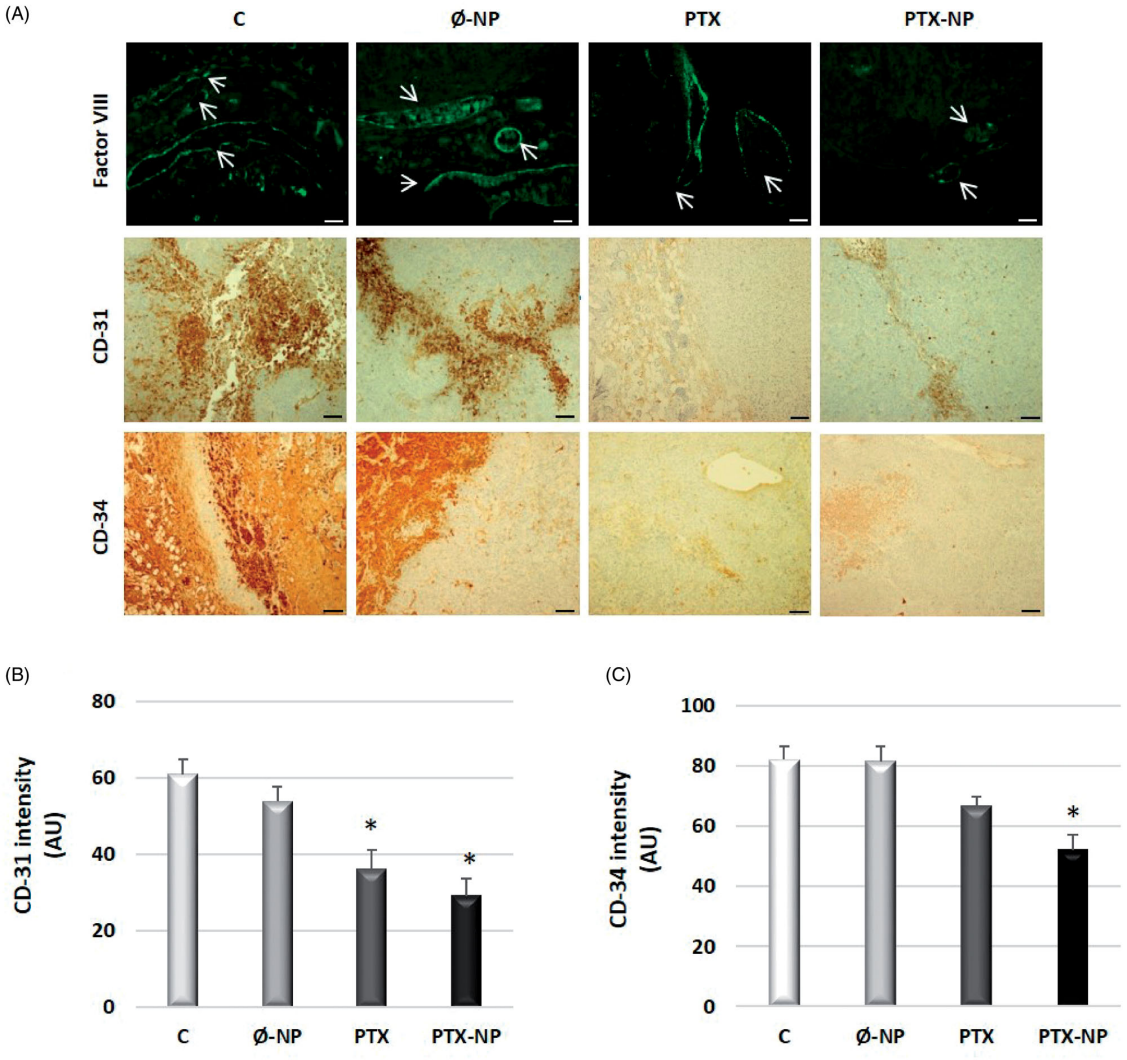
PTX-NP inhibits angiogenesis in the HNSCC tumor model. (A) Measurement of IF and IHC stained Factor VII (top panel), CD31 (central panel), and CD34 (bottom panel) expression in tumors. Scale bar, 100 μm. Representative images were randomly taken blindly. Experimental groups: C (control); Ø-NP (unloaded NPs, 1 mg/ml; PTX (Paclitaxel, 5 mg/kg); and PTX-NP (Paclitaxel loaded NPs, 5 mg/kg for 21 days. (B and C) Quantification of immunostaining for CD-31 (B) and CD-34 (C) using the ImageJ software. Data represent the mean ± SD of arbitrary units (AU). *p < .05 versus C and Ø-NP groups; (*n* = 20).

Finally, we evaluated PTX-NP antiangiogenic potential. Tumor tissue samples were routinely processed and immunohistochemically stained for Factor VIII, CD31, and CD34. The results for Factor VIII [Figure 7(A)] showed that PTX-NP and PTX groups had lower vascular density compared with control and NP-unloaded groups.

The anti-vascular effects of PTX-NP observed in histological sections were confirmed by analysis of CD31 and CD34 antibody staining in mice tumors. CD31 levels decreased significantly after administration of PTX-NP (29.17 ± 4.7 AU) and PTX (36.25 ± 4.6 AU) treatment compared to control (60.85 ± 4.0 AU) [*p* < .05; Figure 7(A,B)]. Similar results were obtained for CD34 but only statistically significant differences between PTX-NP and control groups [*p* < .05; Figure 7(A,C)] were observed. Taken together, all these data indicate that PTX-NP treatment reduces the neovascularization in a murine model of HNSCC.

## Discussion

Concomitant chemotherapy and radiotherapy is the strategy that has proven to be the most effective treatment for patients with a high risk of recurrence. However, systemic cancer chemotherapy is frequently accompanied by strong side effects (Xie et al., 2007). Researchers have shown increased interest in developing selective and tumor-specific drug-delivery vectors, such as polymeric nanoparticles (Xie et al., 2007). Direct intratumoral injecting delivery systems have significant potential advantages due to providing enhanced dosage at the targeted site compared to the systemic approach, reduced side effects toward other organs and tissues, and ease of controlled and sustained drug release administration (Wolinsky et al., 2012; Bu et al., 2019).

In this work, PEG-/B/-POLYMTOS nanoparticles that encapsulate PTX were administered by intratumoral injection in a xenograft mouse model of HNSCC. Our study showed that the average tumor volume as a function of time was lower for the tumor-bearing mice injected with PTX-NP compared to the free PTX intratumoral injection. The inhibitory effects decreased markedly after 2 weeks. It has been demonstrated that the antitumor mechanism of paclitaxel is the inducement of G2/M arrest in the cell cycle, interfering with several signal transduction pathways and inducing apoptosis through the stabilization of microtubules (Trielli et al., 1996). Increased G2/M phase arrest indicates the inhibition of cell division and restraint on cell growth (Shen et al., 2012).

The data obtained on tumor growth are in agreement with the immunohistopathology analysis of the tumor tissue. Tumors collected from mice treated with intratumoral injection of free PTX showed a Ki-67 proliferation index reflecting moderate proliferation, while the tumors from mice treated with PTX-NP had a proliferative index indicating no growth in such cells suggesting that they were exposed to an effective cytotoxic treatment. It is known that the Ki-67 is an antigen expressed in cells that are not in the G0 phase, but in G1, G2, S, and M phases and that tumors with a high Ki-67 proliferative index are rapidly growing and aggressive (Işık Gönül et al., 2008).

Furthermore, we highlight in our study that intratumoral injections of PTX-NP produced a significant inhibitory effect on tumor growth at a dose of paclitaxel of 5 mg/kg, much lower than those administered in other studies. Thus, in contrast to our results, Song et al. (2018) synthesized a nanocarrier system constructed from a hyaluronic acid-disulfide-vitamin E succinate conjugate (HA-SS-VES, HSV) and PTX was loaded in the delivery system. They administered PTX-loaded HSV nanoparticles every 3 days for a total of 18 days at a dose of 10 mg PTX/kg in an A549 mouse xenograft model (lung cancer) demonstrated a great inhibition rate of tumor growth (Song et al., 2018). Moreover, in Hep-2 laryngeal squamous cell carcinoma xenografts, Xie et al (2007), injected paclitaxel-loaded PLGA microspheres intratumorally at 15 and 25 mg/kg resulted in enhanced inhibitory activities against tumor growth and tumor neovascularization (Xie et al., 2007).

The data of the cell apoptosis analysis indicated that PEG-b-polyMTOS nanoparticles could deliver PTX more effectively and rapidly, compared with free PTX, into tumor cells to inhibit tumor growth by the induction of cell apoptosis. The results of the annexin V assay were consistent with data on tumor volume and cell proliferation. Previous studies of Wang et al (2016) on gastric carcinoma cancer cells in both vitro and *in vivo*, showed the pro-apoptotic effect for the combination of Jesridonin and PTX. The apoptotic mechanism induced by Jesridonin plus PTX revealed that the combination therapy synergistically activated the mitochondrial apoptosis pathway (Wang et al., 2016). In addition, Hu et al (2015) suggested that Paclitaxel enhanced apoptosis of the oral cancer cell line, tea8113 (Hu et al., 2015).

Paclitaxel induces apoptosis and inhibits cancer cell proliferation through different signal transduction pathways including, the epidermal growth factor receptor pathway (EGFR) (Li et al., 2012; Hu et al., 2015). In various tumors, such as HNSCC, the EGFR signaling pathway is highly activated and its abnormal activation leads to significant tumor metastasis and cell proliferation in squamous cell carcinoma (Guo et al., 2013; Moreira et al., 2017). Therefore, the selective inhibition of the EGFR signaling pathway has become one of the potential therapeutic targets for future treatment methods for tumors. Thus, we observed that polymeric PTX-nanoparticles, compared to free PTX, decreased EGFR expression in HNSCC tumors. According to the current study, recent reports have shown that Paclitaxel inhibited the growth of the oral cancer cell line, tea8113 malignant proliferation, and enhanced tea8113 cell apoptosis through inhibiting the EGFR signaling pathway (Hu et al., 2015). Also, the results of Ren et al (2015) suggested that poly(ethylene glycol)-di-stearoyl phosphatidylethanolamine (PEG-DSPE) micelles containing paclitaxel are a well-characterized platform for the delivery of paclitaxel to treat EGFR over-expressed laryngeal cancer (Ren et al., 2015).

In the present study, treatment of mice with PTX-NP decrease in the levels of anti-apoptotic proteins Bcl-2 and a simultaneous increase in pro-apoptotic protein p53. The p53 protein is a multifunctional transcription factor, associated with the occurrence and progression of numerous human tumors, mainly responsible for tumor cell apoptosis and cell cycle arrest (Sakashita et al., 2008). Taken together, the above results suggest that PTX-NP induced apoptosis and cell-cycle arrest in the G2/M-phase in FaDu cells of the tumor are likely to be associated with activation of p53 by DNA damage. The Bcl-2 family of proteins, including Bcl-2, Bax, and BH3-interacting domain death agonist, have emerged as regulators of mitochondria-mediated apoptosis (Yang et al., 1997). A previous study revealed that paclitaxel can induce apoptosis of human neuroblastoma cell line (NB-1 cells), which may be mediated by downregulation of Bcl-2 and upregulation of Bax (Nonaka et al., 2006). Regulation of intrinsic apoptosis by Bcl-2 family proteins occurs through ROS production and is followed by a reduction in the MMP level, which stimulates mitochondria to release proapoptotic molecules and results in activation of caspase-9 and -3 (Meshkini and Yazdanparast, 2012).

Cancer cells are characterized by high levels of ROS compared with their normal counterparts (Raza et al., 2017). A moderate increase in ROS promotes cell proliferation, whereas excessive amounts of ROS cause toxicity to cancer cells (Raza et al., 2017). Therefore, manipulation of ROS levels is a promising strategy to selectively kill cancer cells without significant toxicity to normal cells (Schumacker, 2006). The results of the present study demonstrated that levels of ROS and RNS were elevated when PTX-NP was administered, compared to levels of the PTX group. Ren et al (2017) demonstrated that paclitaxel effectively increases ROS levels and reduces SOD levels in an *in vitro* model of canine mammary gland tumor cells (Ren et al., 2018). In human prostate cancer, the combination of paclitaxel and metformin suppressed proliferation and induced apoptosis via ROS, promoting expression of the pro-apoptotic protein p53, and inducing mitochondrial damage (Zhao et al., 2019).

Furthermore, the PEG-/B/-POLYMTOS nanoparticles used in this study are composed of a methacrylic derivative of α-tocopheryl succinate (MTOS). Previously, α-TOS has been shown to induce ROS accumulation in many types of tumor cells. The generation of the mitochondrial permeability transition pore may play a central role in α-TOS induced apoptosis (Dong et al., 2009). In past studies, our group described that α-tocopheryl succinate-based polymeric nanoparticles (poly(VP-co-MTOS nanoparticles) on *in vitro* model of FaDu cells, activated the intrinsic pathway of apoptosis, being related to the accumulation of reactive species, both ROS as RNS (Palao-Suay et al., 2016; Sánchez-Rodríguez et al., 2018). Currently, there is similar research using vitamin E-based nanomedicines for anticancer drug delivery as a treatment for various types of cancer such as melanoma, prostate cancer, or cervical cancer (Wang et al., 2014; Liu et al., 2017; Xia et al., 2018). For example, Wu et al (2015) developed a vehicle for PTX delivery by conjugating polyethylene glycol succinate to D-α-tocopherol (TPGS) and 4-arm polyethylene glycol (4-arm PEG) (Wu et al., 2015). Song et al (2018), developed a redox-sensitive nanocarrier system constructed from a hyaluronic acid-disulfide-vitamin E succinate (HA-SS-VES, HSV) conjugate and PTX was loaded in the delivery system for lung cancer treatment. The antitumor activity results showed that compared to redox-insensitive nanoparticles and PTX, PTX-loaded redox-sensitive nanoparticles exhibited much greater *in vitro* and *in vivo* cytotoxicity and apoptosis-inducing ability against the lung cancer model (Song et al., 2018).

Taken together, these results indicated that PTX-NP induced alterations in the expression of apoptosis-associated proteins and the generation of ROS. However, it remains to be elucidated whether the apoptosis induced by PTX-NP is dependent on mitochondria and is mediated by ROS; the mechanism in which PTX-NP selectively induced apoptosis into a hypopharynx carcinoma squamous cells (FaDu) tumor xenograft mouse model has been defined but requires further investigation.

Also, the present study showed that PTX-NP treated group reduced tumor vascularization compared to the group treated with free PTX, which are presumably related to the elevated sensitivity of vascular endothelial cells. Several studies have demonstrated that paclitaxel has direct effects on endothelial cells (Ng et al., 2004; Huang et al., 2010; Clemente et al., 2019). Paclitaxel can disturb most of the endothelial cell functions involved in angiogenesis since they can inhibit endothelial cell proliferation, motility, and migration (Ng et al., 2004; Huang et al., 2010; Clemente et al., 2019). In this line are the studies by Clemente et al (2019) that investigated the effects of PTX-loaded in pyromellitic nanosponges (PTX-PNS) in reducing *in vivo* melanoma cell growth, invasivity, and in inhibiting angiogenesis. Experiments demonstrated that tumor weights, volumes, and growth were significantly reduced by PTX-PNS treatment concerning PTX; the angiogenesis and the cell proliferation, detected in the tumor samples with CD31 and Ki-67 antibodies, respectively, indicated that, in the PTX-PNS-treated tumors, the tube formation was inhibited, and a low amount of proliferating cells was present (Clemente et al., 2019). Huang et al (2010), evaluated the antiangiogenic activity of sterically stabilized liposomes containing paclitaxel (SSL-PTX) in human breast cancer cells and human breast cancer tumor xenograft nude mice. The *in vitro* results indicated that SSL-PTX effectively inhibits endothelial cell proliferation and migration, and they observed that SSL-PTX inhibited tumor growth in the xenograft model via the antiangiogenic effect (Huang et al., 2010).

## Conclusion

In this study, we have successfully prepared paclitaxel-loaded-PEG-/B/-POLYMTOS nanoparticles for palliative intratumoral injection therapy and evaluated the antitumor efficacy into FaDu squamous cell xenografts. In conclusion, localized delivery of paclitaxel-loaded- nanoparticles resulted in enhanced inhibitory activities against tumor growth and tumor neovascularization without obvious side effects.

The application and development of PEG-/B/-POLYMTOS nanoparticles delivery system for paclitaxel have not been studied in the treatment of HNSCC. Therefore, our nanomedicine strategy could potentially lead to a significant advancement in palliative chemotherapy by intratumoral injection to patients with recurrent or metastatic head and neck squamous cell cancer. All the above findings provide support to carrying out additional preclinical studies on PTX-NPs either as a single agent, possibly at higher doses, or in combination with other drugs in cancer therapy. Future studies will be necessary to provide a clinical approach for human patients.

## Data Availability

Data are available on request from the authors.
